# Supplementary material for: Improving the university teaching-learning process with ECO methodology: Teachers’ perceptions
Source: PLoS One. 2020 Aug 18;15(8):e0237712. doi: 10.1371/journal.pone.0237712 (PMC7433871; doi:10.1371/journal.pone.0237712)
Supplement: S1 Questionnaire — (PDF) [file pone.0237712.s001.pdf]

QUESTIONNAIRE ABOUT ECO METHOD IMPLEMENTATION IN THE UNIVERSITY

The purpose of this questionnaire is to know the impact of the implementation of the ECO method in the University. It is part of an innovation project, entitled *ECO in Higher Education. Teaching inspired by the environment*, funded by the University of Seville's 3rd Teaching Plan. It is intended for teachers who have been part of the innovation project. The data collected will be analysed to be used in the final report, as well as in some conference communications or journal articles.

The questionnaire consists of 29 questions, divided into three blocks: 12 initial questions on demographic data, 9 closed-ended questions with four options and 8 open-ended questions. For open-ended questions, we ask that you answer in detail and as explicitly as possible.

Each teacher is requested to complete one questionnaire per course in which they have implemented the ECO method. Similarly, if there have been two teachers in a shared course, each must fulfill, from their experience, a separate questionnaire.

Thank you very much for your collaboration.

Prof. Juan-Jesús Torres-Gordillo
University of Seville (Spain)

DEMOGRAPHIC QUESTIONS BLOCK

1. Gender

Male

Female

2. Age

[ ]

3. Years of experience as university teacher

[ ]

4. Years of experience as a teacher at other educational levels (non-university)

[ ]

5. Select your University

Sevilla

Barcelona

Vigo

Gran Canaria

Buenos Aires/La Plata

Other: ________________

6. Write the name of the course in which you have implemented the ECO method
(remember that you must fulfill a questionnaire for each course you have implemented with ECO)*

[ ]

7. Course type
1. Basic education
2. Mandatory
3. Optional
4. Other type (Free organization courses by US or similar)

8. Class shift
1. Morning
2. Afternoon

9. Quarter/semester
1. 1st
2. 2nd

10. Academic year
1. 1st
2. 2nd
3. 3rd
4. 4th
5. Master
6. PhD

11. Knowledge areas
1. Science
2. Engineering/Architecture
3. Health Sciences
4. Social and Legal Sciences
5. Arts and Humanities

12. Courses/years of experience applying the ECO method
1. 18-19 (first year applying ECO)
2. From 17-18 (two courses applying ECO)
3. From 16-17 (three courses applying ECO)
4. More than 3 years applying ECO

BLOCK OF CLOSED-ENDED QUESTIONS ABOUT THE ECO METHOD

13. I applied ECO to:

1	One specific topic or less	3	A complete course
2	A thematic block	4	Other

14. Initial real expectations regarding improvement of the teaching-learning process using ECO
1. Low
2. Medium
3. High
4. Very high

15. Difficulties encountered with ECO
1. Many (overwhelmed)
2. Several (not easily assumed)
3. Some (assumable)
4. None (or minor ones)

16. Benefits found by applying ECO
1. None (or minor ones)
2. Some (recognizable)
3. Several highlights (recognizable)
4. Many (very positive overall)

17. Observed impact on professional development as a teacher
1. Low
2. Medium
3. High
4. Very high

18. Observed impact on student learning
1. Low
2. Medium
3. High
4. Very high

19. Observed impact on the improvement of the course
1. Low
2. Medium
3. High
4. Very high

20. Satisfaction with your students' learning outcomes
1. Low
2. Medium
3. High
4. Very high

21. Overall satisfaction with the application of the ECO method
1. Low
2. Medium
3. High
4. Very high

BLOCK OF OPEN-ENDED QUESTIONS ABOUT THE ECO METHOD

We request that these questions be answered in detail and with explicit data. The questions ask about the benefits and difficulties at different levels, separately: teacher, student, teacher-student interaction, didactic process or course design, classroom/team/group management, and assessment process.

22. Main benefits and difficulties found applying ECO as a teacher

23. Main benefits and difficulties encountered applying ECO at the student level

24. Main benefits and difficulties encountered applying ECO at the teacher-student interaction/communication

25. Main benefits and difficulties encountered when applying ECO at the didactic process or design level of the course/materials

26. Main benefits and difficulties encountered applying ECO at classroom, team or group management level

27. Main benefits and difficulties encountered applying ECO at the level of the assessment process (continuous attention to learning/student/ course improvement, not to be confused with grading)

28. In short, what is the main contribution and the main difficulty of ECO?

29. Main changes made in the course to apply ECO with respect to previous years with another methodology
