# Supplementary material for: Improving the university teaching-learning process with ECO methodology: Teachers’ perceptions
Source: PLoS One. 2020 Aug 18;15(8):e0237712. doi: 10.1371/journal.pone.0237712 (PMC7433871; doi:10.1371/journal.pone.0237712)
Supplement: Attachment — Submitted filename: _Response to Reviewers.docx [file pone.0237712.s002.docx]

**Response to Reviewers**

PONE-D-19-34682

Improving the university teaching-learning process with ECO methodology: Teachers’ perceptions

PLOS ONE

**Response to Journal requirements**

| **Editor's comments** | **List of changes of each point that has been raised** |
| --- | --- |
| 1. Please ensure that your manuscript meets PLOS ONE's style requirements, including those for file naming. The PLOS ONE style templates can be found at  [http://www.journals.plos.org/plosone/s/file?id=wjVg/PLOSOne_formatting_sample_main_body.pdf](http://www.journals.plos.org/plosone/s/file?id=wjVg/PLOSOne_formatting_sample_main_body.pdf) and [http://www.journals.plos.org/plosone/s/file?id=ba62/PLOSOne_formatting_sample_title_authors_affiliations.pdf](http://www.journals.plos.org/plosone/s/file?id=ba62/PLOSOne_formatting_sample_title_authors_affiliations.pdf) | PLOS ONE's style requirements has been thoroughly revised. |
| 2. PLOS ONE will consider submissions that present new methods, software, or databases as the primary focus of the manuscript if they meet the criteria of utility, validation, and availability described here: <http://journals.plos.org/plosone/s/submission-guidelines#loc-methods-software-databases-and-tools>. To meet these criteria, please provide supporting materials enabling other teachers and researchers to replicate your teaching intervention such as sample worksheets, a detailed lesson plan or curriculum or other educational materials. If you include supporting materials, they should not be under a copyright more restrictive than CC-BY.” | The instrument used in the research has been incorporated as complementary material. This document has been translated into English. See [[dx.doi.org/10.17504/protocols.io.bhp5j5q6](https://dx.doi.org/10.17504/protocols.io.bhp5j5q6)] |
| 3. Thank you for including your ethics statement:  'All participants in this research knew the nature of the study and the conditions of their participation. This participation was voluntary and followed the rules of informed consent. This research has followed the internal regulations in the field of Social Sciences of the Ethical Committee of Experimentation of the University of Seville (Spain).'  Please amend your current ethics statement to confirm that your named institutional review board or ethics committee specifically approved this study.  Once you have amended this/these statement(s) in the Methods section of the manuscript, please add the same text to the “Ethics Statement” field of the submission form (via “Edit Submission”).  For additional information about PLOS ONE ethical requirements for human subjects research, please refer to <http://journals.plos.org/plosone/s/submission-guidelines#loc-human-subjects-research>. | This information has been incorporated into the text. The authors have added the following text into the main paper document:  “On top of that, this research was approved by the Ethical Committee of Experimentation in Social Sciences of the University of Seville and followed its standards”.  The authors have added this text to the “Ethics Statement” field of the submission form, by rewriting the text as follows:  “This research has followed the internal regulations in the field of Social Sciences and was approved by the Ethical Committee of Experimentation of the University of Seville (Spain).” |

**COMMENTS TO THE AUTHOR**

**Responses to the reviewer #2**

| **Your comments** | **List of changes of each point that has been raised** |
| --- | --- |
| I have only very few minor suggestions for further improvement.  1. Abstract: There is no need to explain what ECO is. | The authors have removed this comment from the abstract:  “ECO is an inductive methodology based on challenges and inspired by Design Thinking”, following the reviewer's recommendations. |
| 2. The Introduction is not well-written. The authors uncritically reviewed previous studies without pointing out the research gap in the existing literature. Why the present study has to be conducted? How will the present study address the gap surfaced in previous studies? In other words, the rationale for the present study is not clearly stated. | The authors have reviewed the Introduction section. The modifications of the text have been highlighted in the text in grey. The authors have incorporated the following texts to the main paper document:  “Although there are innovative experiences as we have mentioned, our review has not found methodological innovations that are really transforming what happens in the learning process, as ECO tries to do, and especially from a committed and conscious work of the students. In addition, another gap in the literature is that there are few studies that focus on analyzing teachers' views and perceptions and how they feel and interpret what is happening in the classroom. We want to test the ECO method and fill this gap.” |
| 3. Since questionnaire is the most important instrument for the present study, I was wondering whether the reliability of the questionnaire was checked. The authors have to state the reliability of the questionnaire. Please also include the full questionnaire in supplementary materials. | The instrument used in the research has been incorporated as complementary material. This document has been translated into English. Also, the authors have incorporated the following text to the Instrument section: [[dx.doi.org/10.17504/protocols.io.bhp5j5q6](https://dx.doi.org/10.17504/protocols.io.bhp5j5q6)]  The reliability of the questionnaire was checked and incorporated into the Method section. The modifications of the text have been highlighted in the text in grey. The authors have incorporated the following text to the main paper document:  “Cronbach’s alpha coefficient for this instrument was 0,853.” |
| 4. The Discussion section is anything but substantial. The authors did not relate the present findings with previous studies. | The authors have reviewed the Discussion section. The modifications of the text have been highlighted in the text in grey. The authors have incorporated the following texts to the main paper document:  “The results of active teaching methods are usually evaluated through the students' own testimonies, which is why studies with more in-depth techniques are necessary [43]. Therefore, the objectives of this study were to describe university teaching staff’s perceptions of the impact made by the ECO method: firstly, on teaching; and secondly, on student learning. In this way, teachers’ perspectives as agents involved in the teaching-learning process could be highlighted. “  “With regard to the first objective, we can conclude that the results show the implementation of the ECO method to have a very positive impact on teachers in terms of their personal and emotional development [25].”  “When teachers manage to gain the necessary autonomy, they are able to undertake innovative methodologies. In this sense, other research [45-46], agrees in pointing out how this autonomy that teachers acquire is what leads them to participate in innovative initiatives and projects that promote their personal development from the educational context.”  “This conclusion is also shared by Torres-Gordillo & Herrero-Vázquez [47], who point out how teachers, in their search to improve teaching and students' professional development, satisfactorily value the innovations they make according to the impact they observe in learning and in quality improvement of their courses.”  “The teacher gives a more central role to the students, and takes on the role of facilitator and companion, generating new dynamics in the classroom, as reflected in other studies [45, 48-49].”  “This collaborative work between teachers, as asserted in similar research [50-52], allows teachers to express their doubts and reflect together on the progress made, receiving continuous feedback and feedforward, as well as sharing resources to improve teaching.”  “This idea is shared by Ayllón, Alsina & Colomer [54], whose research states that students obtain higher grades when their teachers show them confidence and willingness by offering them the necessary academic help and resources.”  “With regard to the second objective, the results lead us to conclude that the ECO method helps to transform students, providing them with a more well-rounded and socially-aware education, as other studies indicate [3,11,22]. An education in which the development of competencies is especially present.”  “The method’s most important contribution is its ability to develop the creativity of students, as well as their interest in undertaking projects and providing original, unique and authentic solutions, as reflected in other research [59-60].”  “This promotes a dynamic of teamwork on which it is based, where students are required to develop empathy, as well as engaging in dialogue and conflict resolution, as considered in other research [62-63]. In this way, through ECO, students become more autonomous and responsible for completing tasks, both individually and in groups, committing them to the process [64]. Students come to understand that effort and rigour are integral parts of engaging in the working process, providing them with great satisfaction in achieving results and final products, in which other studies coincide [65-67].”  “It encourages students to connect with them, learn from them, and to look for real solutions and proposals, as other research indicates [68-69]. Furthermore, it allows students to visualise themselves professionally [70], connect with professionals from their field of knowledge, and become familiar with the competencies that they will exercise in their professional environment. This is highly valued by students, as reflected in the study by Villalobos-Abarca, Herrera-Acuña, Ramírez, & Cruz [71]. The ECO method helps develop civic and social commitment in students, providing them with a more socially-responsible education. This aspect is shared by other studies [22, 72-74], which stress the need to go further, and urge the university to commit to the development of innovative practices with society, transferring its knowledge, strategies and values.”  “Finally, we conclude by pointing out the importance of promoting refreshing teaching methodologies, transformative practices and innovative projects, due to their positive impact on the quality and relevance of university education [72]. And, above all, because they manage to awaken the motivation of students, one of the main goals of any university teacher [75].”  Also, we have added 17 new references:   43 Hartikainen S, Rintala H, Pylväs L, Nokelainen P. The concept of active learning and the measurement of learning outcomes: A review of research in engineering higher education. Educ. Sci. 2019; 9(4):276. <http://dx.doi.org/10.3390/educsci9040276> 45 Gavrilyuk OA, Tareva EG, Lakhno AV. Investigating the association between university teachers’ professional autonomy and their innovation performance. Pedagogika. 2019; 133(1):128–148. <http://dx.doi.org/10.15823/p.2019.133.7> 46 Kumpulainen K, Vierimaa SM, Koskinen-Koivisto E. Developing connective pedagogy in cultural research–A case study from the teachers’ perspective in adopting a problem-based approach in higher education. Educ. Sci. 2019; 9(4):252. <http://dx.doi.org/10.3390/educsci9040252> 47 Torres-Gordillo JJ, Herrero-Vázquez EA. Innovación metodológica transdisciplinar en la universidad con el método ECO. In: Reyes-Tejedor M, Cobos-Sanchiz D, López-Meneses E, Coords. Innovación pedagógica universitaria: reflexiones y estrategias. Barcelona: Octaedro; 2020. Cap. 9 50 Dawson P, Henderson M, Mahoney P, Phillips M, Ryan T, Boud D, et al. What makes for effective feedback: staff and student perspectives. Assess Eval High Educ. 2018; 44(1):25–36. <https://www.doi.org/10.1080/02602938.2018.1467877> 51 Kates FR, Samuels SK, Case JB, Dujowich M. Lessons Learned from a pilot study implementing a team-based messaging application (Slack) to improve communication and teamwork in Veterinary Medical Education. J Vet Med Educ. 2020; 47(1):18–26. https://www.doi.org/10.3138/jvme.0717-091r2   52 Torres-Gordillo JJ, García-Jiménez J, Herrero-Vázquez, EA. Contributions of technology to cooperative work for university innovation with Design Thinking [Aportaciones de la tecnología al trabajo cooperativo para la innovación universitaria con Design Thinking]. Píxel-Bit. 2020; 59. <http://dx.doi.org/10.12795/pixelbit.74554>   54 Ayllón S, Alsina Á, Colomer J. Teachers’ involvement and students’ self-efficacy: Keys to achievement in higher education. Dalby AR, Editor. PLoS ONE. 2019; 14(5). <http://dx.doi.org/10.1371/journal.pone.0216865> 55 Rodríguez-Martínez A, Cortés-Pascual A, Val-Blasco S. Análisis de la mejora del nivel de empleabilidad de los universitarios mediante la mejora de competencias transversales y habilidades [Analysis of the increase of the employability level in university students through the improvement of transversal]. Rev. Española Orientac. Psicopedag. 2019; 30(3):102-119. <http://dx.doi.org/10.5944/reop.vol.30.num.3.2019.26275> 58 Sá MJ, Serpa S. Transversal competences: their importance and learning processes by higher education students. Educ. Sci. 2018; 8(3):126. <http://dx.doi.org/10.3390/educsci8030126> 59 Safapour E, Kermanshachi S, Taneja P. A review of nontraditional teaching methods: Flipped classroom, gamification, case study, self-learning, and social media. Educ. Sci. 2019; 9(4):273. <http://dx.doi.org/10.3390/educsci9040273>   64 Jeong J, Cañada-Cañada F, González-Gómez D. The study of flipped-classroom for pre-service science teachers. Educ. Sci. 2018; 8(4):163. <http://dx.doi.org/10.3390/educsci8040163>   67 Valūnaitė-Oleškevičienė G, Puksas A, Gulbinskienė D, Mockienė L. Student experience on the development of transversal skills in university studies. Pedagogika. 2019; 133(1):63–77. <http://dx.doi.org/10.15823/p.2019.133.4> 71 Villalobos-Abarca MA, Herrera-Acuna, RA, Ramírez, IG, Cruz, XC. Aprendizaje basado en proyectos reales aplicado a la formación del ingeniero de software [Real project-based learning applied to software engineers’ education]. Form. Univ. 2018; 11(3):97-112.  <http://dx.doi.org/10.4067/S0718-50062018000300097> 72 Pérez-Pérez C, González-González H, Lorenzo-Moledo M, Crespo-Comesaña J, Belando-Montoro MR, Costa-París A. Service-learning in Spanish universities: A study based on the perception of the dean's teams [Aprendizaje-Servicio en las universidades españolas: un estudio basado en la percepción de los equipos decanales]. RELIEVE. 2019; 25(2). <http://dx.doi.org/10.7203/relieve.25.2.15029> 73 Warren, S. Re-Thinking the “Problem” in Inquiry-Based Pedagogies through Exemplarity and World-Oriented. Educ. Sci. 2019; 9(4):295. <http://dx.doi.org/10.3390/educsci9040295> 75 Herrero-Vázquez EA, Torres-Gordillo JJ. ECO en la educación superior: un aprendizaje resonante. In: Reyes-Tejedor M, Cobos-Sanchiz D, López-Meneses E, Coords. Innovación pedagógica universitaria: reflexiones y estrategias. Barcelona: Octaedro; 2020. Cap. 8. |
